# Supplementary material for: Mechanical signaling through membrane tension induces somal translocation during neuronal migration
Source: EMBO J. 2024 Dec 20;44(3):767–80. doi: 10.1038/s44318-024-00326-8 (PMC11790904; doi:10.1038/s44318-024-00326-8)
Supplement: Supplementary file 1 — Appendix [file 44318_2024_326_MOESM1_ESM.pdf]

Appendix for

Mechanical signaling through membrane tension induces somal translocation during neuronal migration

Table of contents:

Appendix Figure S1 (page 2)
Appendix Figure S2 (page 3)
Appendix Figure S3 (page 4)
Appendix Figure S4 (page 5)
Appendix Figure S5 (page 6)
Appendix Figure S6 (page 7)
Appendix Figure S7 (page 9)
Appendix Figure S8 (page 10)

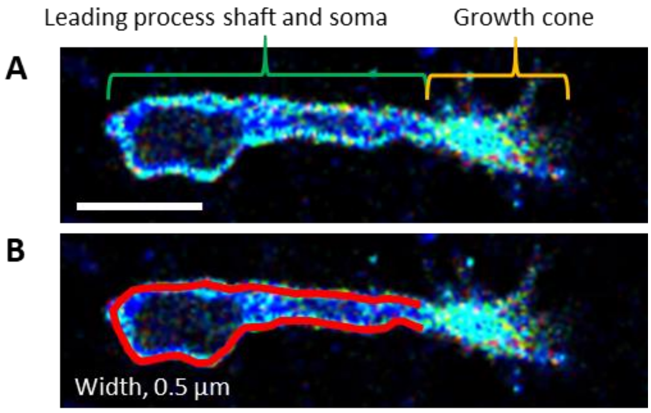

Appendix Figure S1. Regions of the olfactory interneuron analyzed by Flipper-TR. A fluorescence lifetime image of Flipper-TR in an olfactory interneuron migrating in 3D Matrigel. The image is the reuse of Fig. 1A.

(A) We defined the tip of the leading process with a palm-like morphology bearing filopodia and lamellipodia as the growth cone (yellow), and the remaining regions of the cell as the leading process shaft and soma (green). Scale bar, 10 µm.

(B) To analyze the Flipper-TR signal in the leading process shaft and soma, a 0.5 µm wide line was drawn along the plasma membrane region (red line) and the averaged fluorescence lifetime of Flipper-TR was measured on the line.

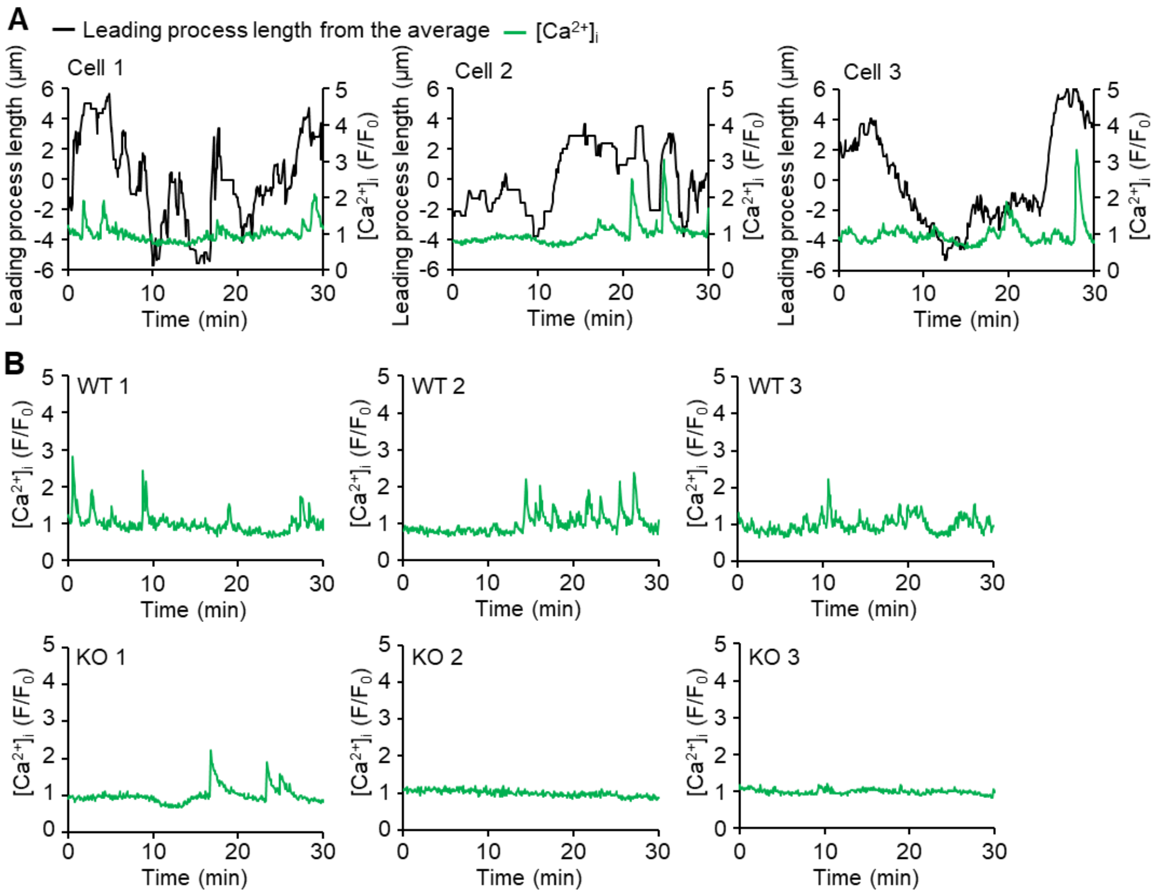

Appendix Figure S2. Additional data of Fig. 2B and F.

(A) Time courses of the leading process length and $[Ca^{2+}]_i$ of migrating olfactory interneurons. Neurons were imaged at 5-sec intervals for 30 min. The average leading process length was set as 0. See also Fig. 2B.

(B) Time courses of $[Ca^{2+}]_i$ in migrating WT (upper) and shootin1 KO (lower) olfactory interneurons. Neurons were imaged at 5-sec intervals for 30 min. See also Fig. 2F.

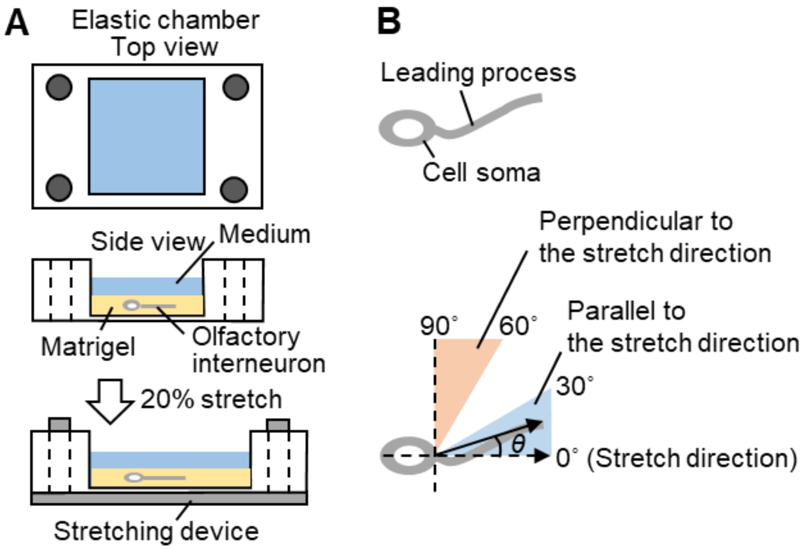

Appendix Figure S3. Schematic diagrams of cell stretching assay.

(A) Olfactory interneurons were suspended in Matrigel, and plated on elastic chambers. The chamber was set on the stretching device, and stretched uniaxially (20% stretch).

(B) The definition of cell orientation in this study. We measured the angle of the leading process with respect to the stretch direction (θ). When the angle was between 0° and 30°, we determined that the leading process was extended (parallel extension). On the other hand, when the angle was between 60° and 90°, the stretch direction was determined to be perpendicular to the leading process.

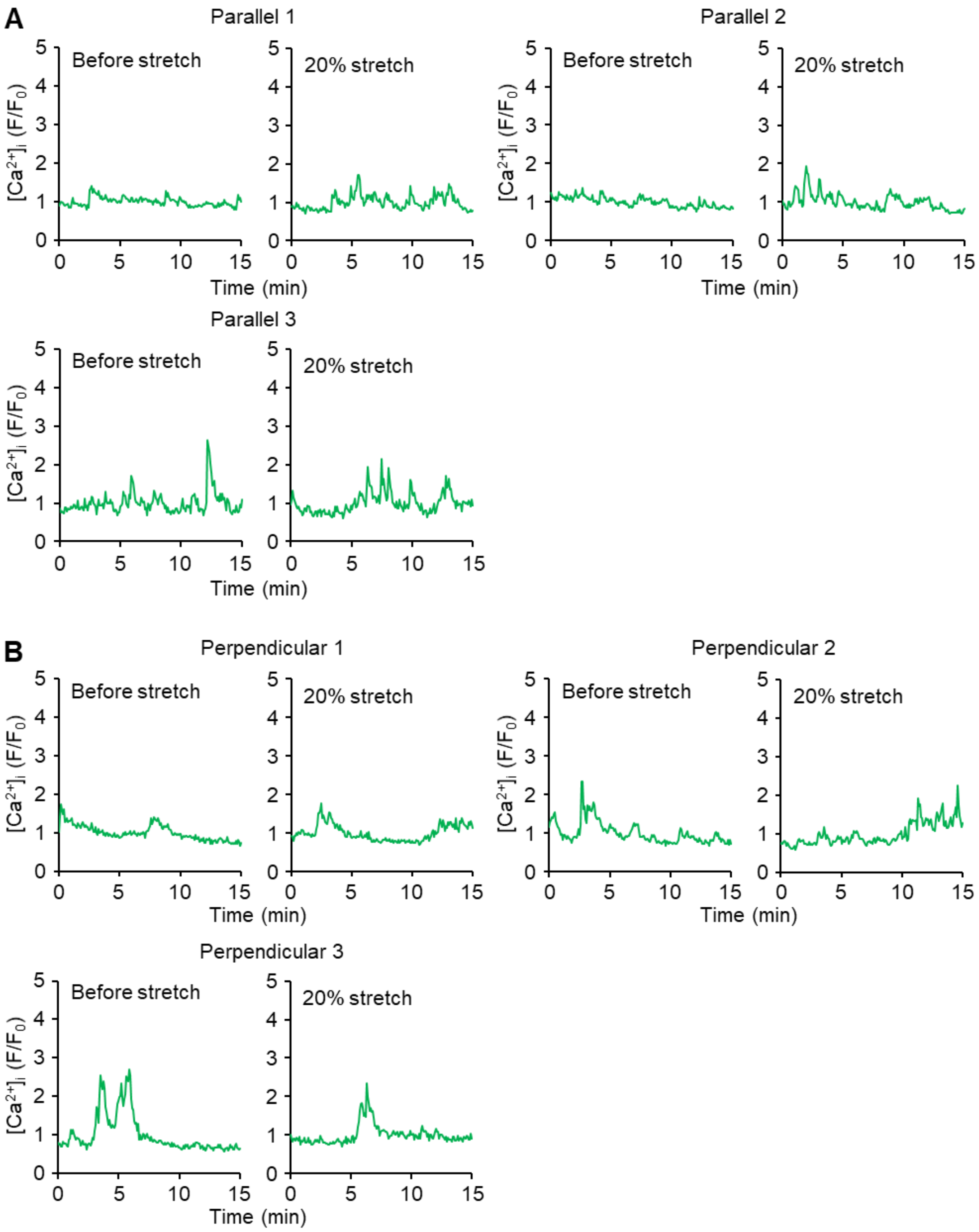

Appendix Figure S4. Additional data of Fig. 2H and I.

Time courses of $[Ca^{2+}]_i$ of migrating olfactory interneurons before and after 20% stretch. We selected migrating olfactory interneurons with leading processes oriented parallel (A) or perpendicular (B) to the stretch direction. Neurons were imaged at 5-sec intervals for 15 min before and after 20% stretch. See also Fig. 2H and I.

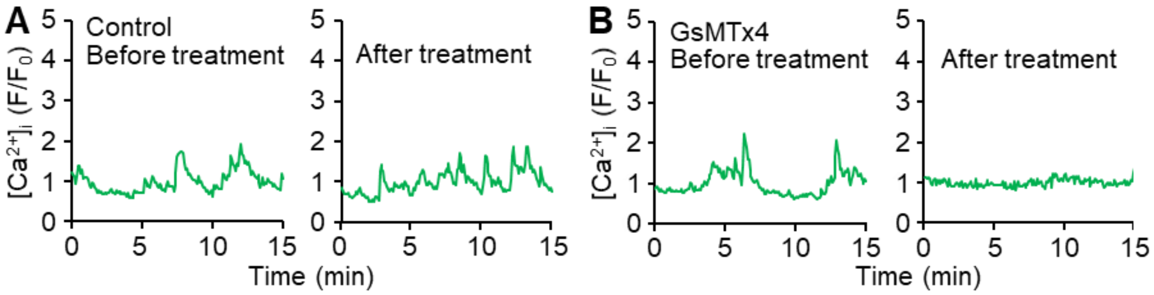

Appendix Figure S5. Inhibition of mechanosensitive channels reduces Ca^{2+} transients of migrating olfactory interneurons.

(A and B) Time courses of $[Ca^{2+}]_i$ in migrating olfactory interneurons treated with vehicle control (A) or 5 µM GsMTx4 (B). Migrating neurons were imaged at 5-sec intervals for 15 min before and after treatment. See also Fig. 3A.

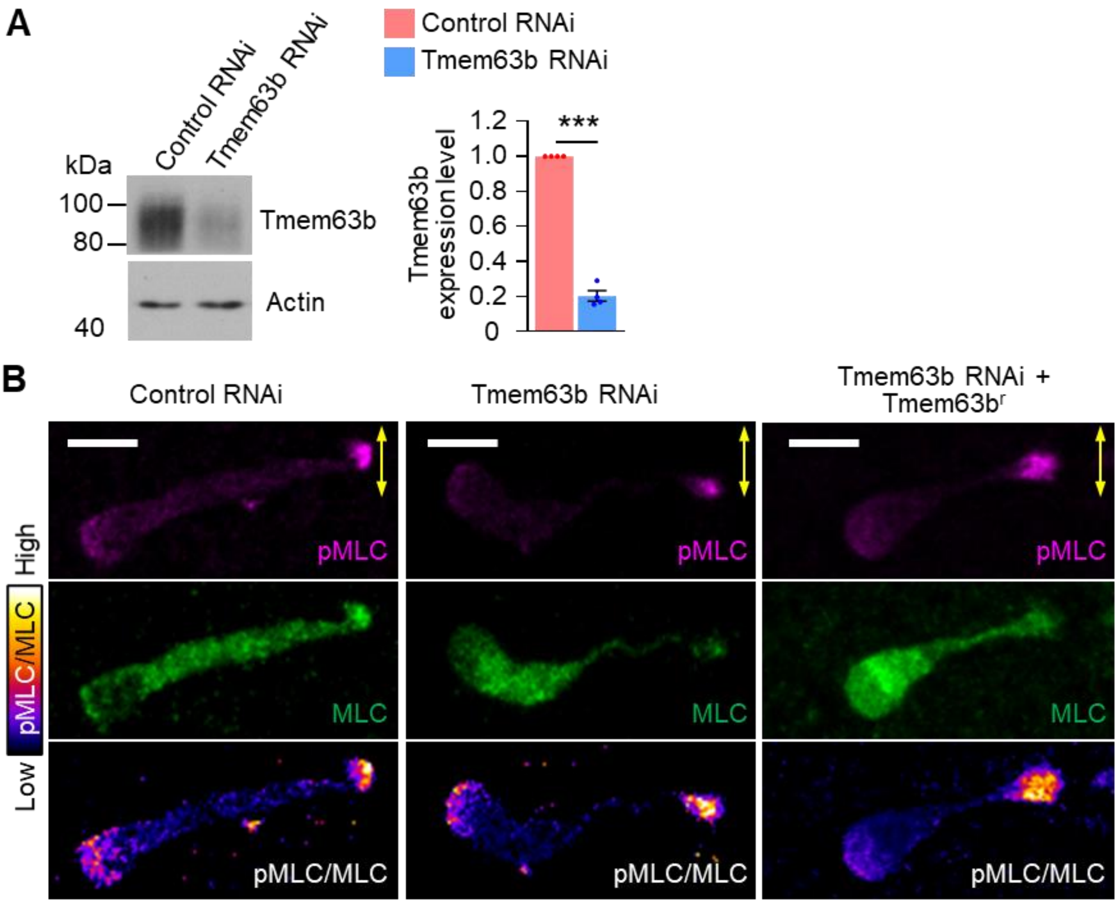

Appendix Figure S6. Analyses of extension-induced Ca^{2+} transients and myosin II activity in olfactory interneurons expressing control microRNA, Tmem63b microRNA or Tmem63b microRNA + Tmem63b^{r}.

(A) Immunoblot analysis of Tmem63b in olfactory interneurons expressing control microRNA or Tmem63b microRNA. Anti-actin antibody served as loading controls. The graph shows the Tmem63b expression levels. The level of Tmem63b in olfactory interneurons expressing control microRNA was normalized as 1. n = 4 independent experiments. Data represent means ± SEM. Statistical analysis was performed using the two-tailed unpaired Welch's t-test. ***p = 0.00012.

(B) Fluorescence images of olfactory interneurons expressing control microRNA, Tmem63b microRNA or Tmem63b microRNA + Tmem63br. Olfactory interneurons cultured on elastic chambers were fixed after 20% stretch, and then stained with anti-phospho-myosin light chain 2 (pMLC) antibody (magenta), anti-myosin light chain 2 (MLC) antibody (green) and DAPI. The ratio of the fluorescence intensity of pMLC to

that of MLC (pMLC/MLC) was displayed by the pseudocolor bar. Neurons with leading processes oriented perpendicular to the stretch direction (yellow arrows) were analyzed as controls of the data in Fig. 4B. Scale bars, 10 μm.

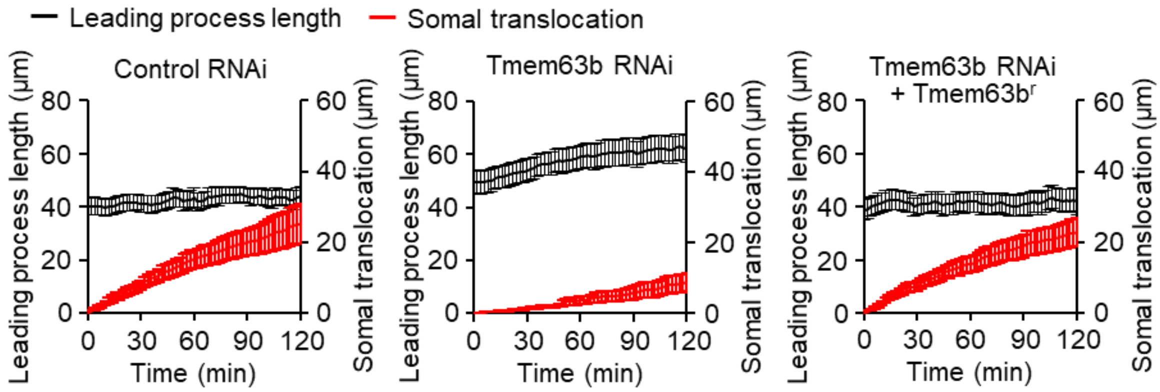

Appendix Figure S7. Average leading process length (black) and average somal translocation (red) of olfactory interneurons expressing control microRNA, Tmem63b microRNA or Tmem63b microRNA + Tmem63b^{r} in Fig. 5A. Control microRNA, n = 19 cells; Tmem63b microRNA, n = 21 cells; Tmem63b microRNA + Tmem63br, n = 18 cells. Data represent mean ± SEM.

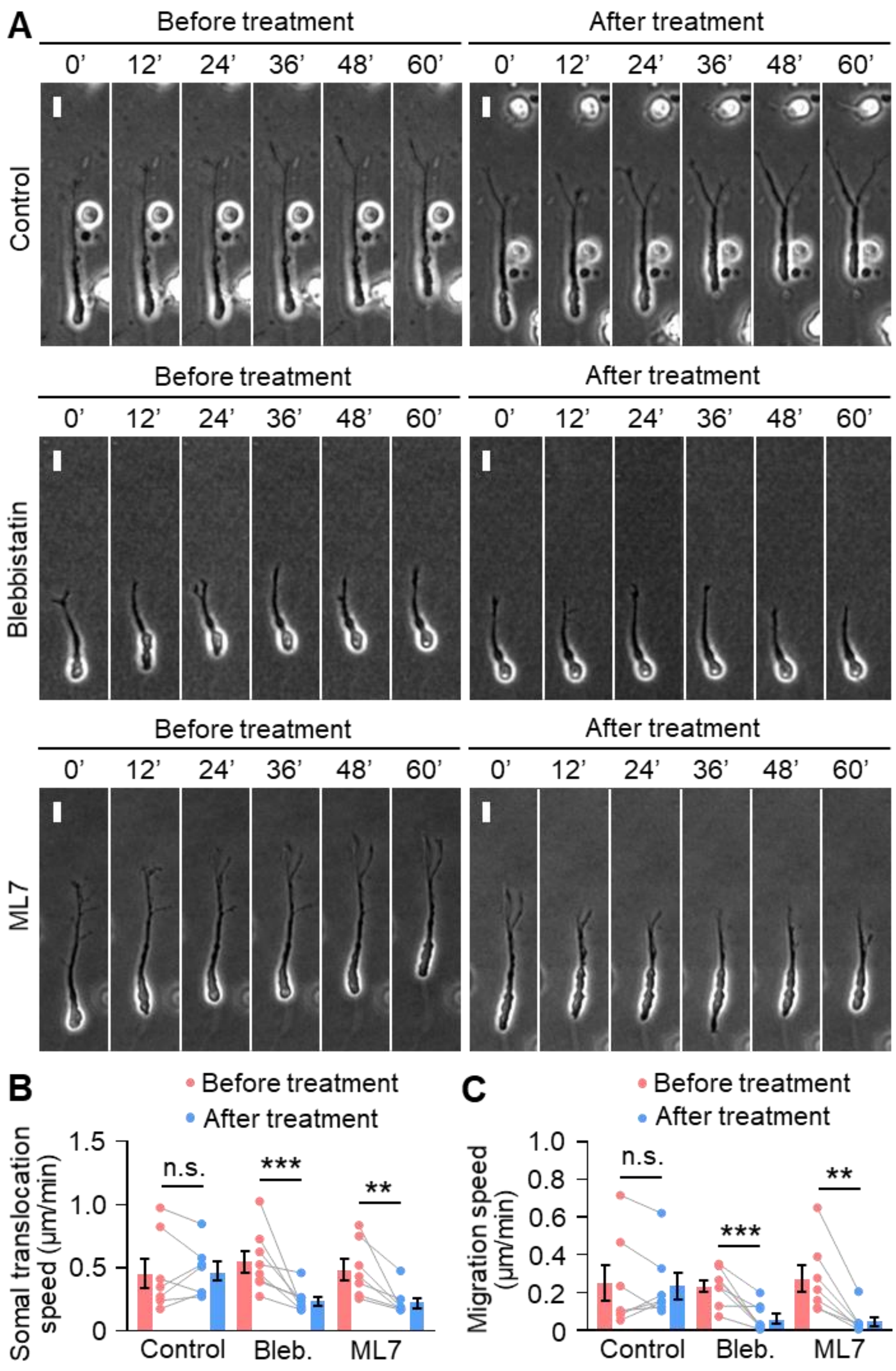

Appendix Figure S8. Inhibition of myosin II or myosin light chain kinase decreases somal translocation and migration speeds of olfactory interneurons.

(A) Time-lapse images of migrating olfactory interneurons before and after treatment with DMSO (control), 100 μM blebbistatin or 10 μM ML7. Neurons were imaged at 2-min intervals.

(B and C), Somal translocation speed (B) and migration speed (C) of migrating olfactory interneurons treated with DMSO (control), 100 µM blebbistatin (bleb.) or 10 µM ML7 in (A). Control, $n = 7$ cells; blebbistatin, $n = 8$ cells; ML7, $n = 7$ cells. (B) $p = 0.938$ (control); 0.00781 (blebbistatin); $p = 0.0156$ (ML7). (C) $p = 0.813$ (control); 0.00781 (blebbistatin); $p = 0.0156$ (ML7). Data represent means ± SEM. Two-tailed Wilcoxon signed-rank test (B and C). **$p < 0.02$; ***$p < 0.01$; n.s., not significant. Scale bars, 10 µm.
