# Supplementary material for: Mechanical signaling through membrane tension induces somal translocation during neuronal migration
Source: EMBO J. 2024 Dec 20;44(3):767–80. doi: 10.1038/s44318-024-00326-8 (PMC11790904; doi:10.1038/s44318-024-00326-8)
Supplement: Supplementary file 4 — Movie EV1 [file 44318_2024_326_MOESM4_ESM.zip › Movie EV1/Movie EV1 legend.docx]

**Movie EV1. Time-lapse movies of DIC (left) and [Ca^2+^]_i_ (right) of a migrating olfactory interneuron loaded with 1 µM CalRed R525/650 (see Fig. 2A,B).** Images were acquired at 5-sec intervals for 30 min. Scale bar, 10 µm.
