# Supplementary material for: Mechanical signaling through membrane tension induces somal translocation during neuronal migration
Source: EMBO J. 2024 Dec 20;44(3):767–80. doi: 10.1038/s44318-024-00326-8 (PMC11790904; doi:10.1038/s44318-024-00326-8)
Supplement: Supplementary file 5 — Movie EV2 [file 44318_2024_326_MOESM5_ESM.zip › Movie EV2/Movie EV2 legend.docx]

**Movie EV2. A time-lapse movie of [Ca^2+^]_i_ (fire) of migrating olfactory interneuron loaded with 1 µM CalRed R525/650 (see Fig. 2H).** The neuron was cultured on an elastic chamber and exposed to 20% stretch (yellow arrows). Images were acquired at 5-sec intervals for 15 min before and after 20% stretch. Scale bar, 10 µm.
