# Supplementary material for: Mechanical signaling through membrane tension induces somal translocation during neuronal migration
Source: EMBO J. 2024 Dec 20;44(3):767–80. doi: 10.1038/s44318-024-00326-8 (PMC11790904; doi:10.1038/s44318-024-00326-8)
Supplement: Supplementary file 6 — Movie EV3 [file 44318_2024_326_MOESM6_ESM.zip › Movie EV3/Movie EV3 legend.docx]

**Movie EV3. Time-lapse movies of migrating olfactory interneuron expressing control microRNA (left), Tmem63b microRNA (middle) and Tmem63b microRNA + Tmem63b^r^ (right) (see Fig. 5A,B).** Phase contrast images were acquired at 2-min intervals for 120 min. Scale bar, 10 µm.
