# Supplementary figures and images for: Mechanical signaling through membrane tension induces somal translocation during neuronal migration
Source: EMBO J. 2024 Dec 20;44(3):767–80. doi: 10.1038/s44318-024-00326-8 (PMC11790904; doi:10.1038/s44318-024-00326-8)

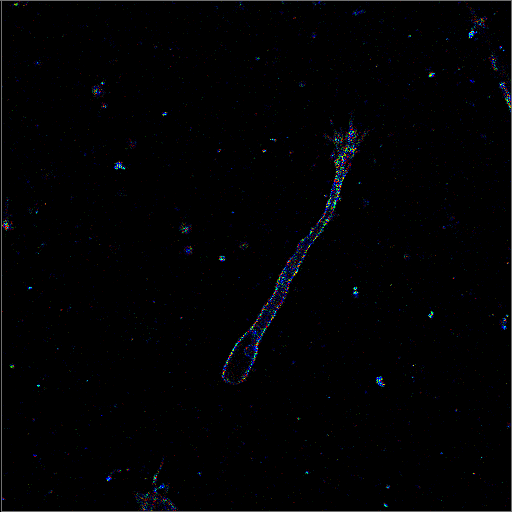

Supplement: Supplementary file 7 — Source data Fig. 1 [file 44318_2024_326_MOESM7_ESM.zip › Fig.1/Fig. 1A/Lower.tif]

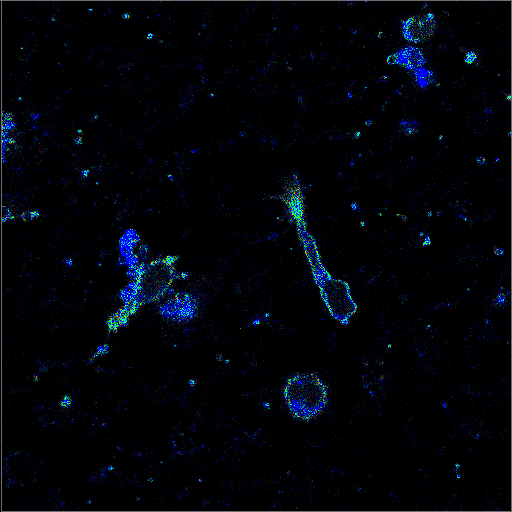

Supplement: Supplementary file 7 — Source data Fig. 1 [file 44318_2024_326_MOESM7_ESM.zip › Fig.1/Fig. 1A/Upper.tif]

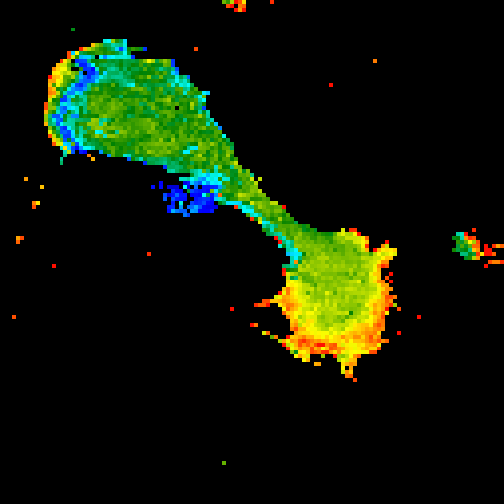

Supplement: Supplementary file 7 — Source data Fig. 1 [file 44318_2024_326_MOESM7_ESM.zip › Fig.1/Fig. 1C.bmp]

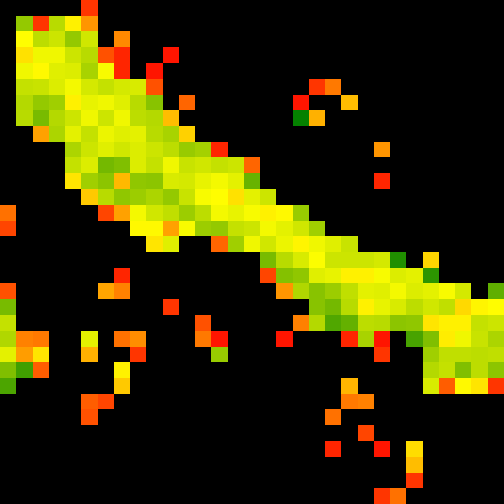

Supplement: Supplementary file 7 — Source data Fig. 1 [file 44318_2024_326_MOESM7_ESM.zip › Fig.1/Fig. 1E/Lower_0 h.bmp]

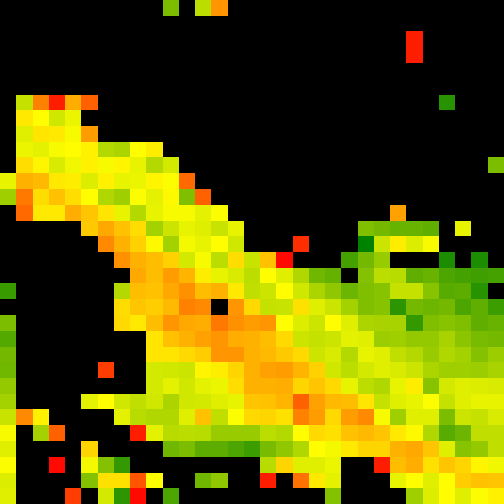

Supplement: Supplementary file 7 — Source data Fig. 1 [file 44318_2024_326_MOESM7_ESM.zip › Fig.1/Fig. 1E/Lower_1 h.bmp]

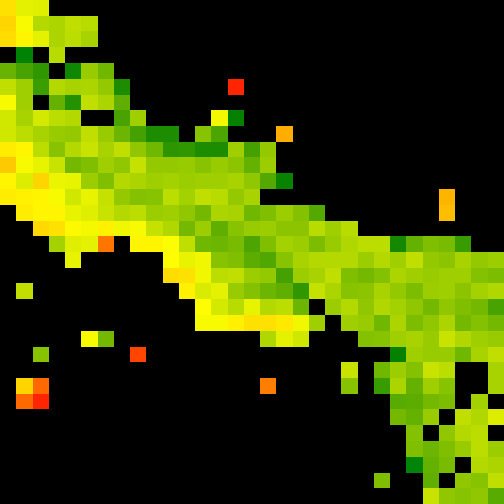

Supplement: Supplementary file 7 — Source data Fig. 1 [file 44318_2024_326_MOESM7_ESM.zip › Fig.1/Fig. 1E/Lower_2 h.bmp]

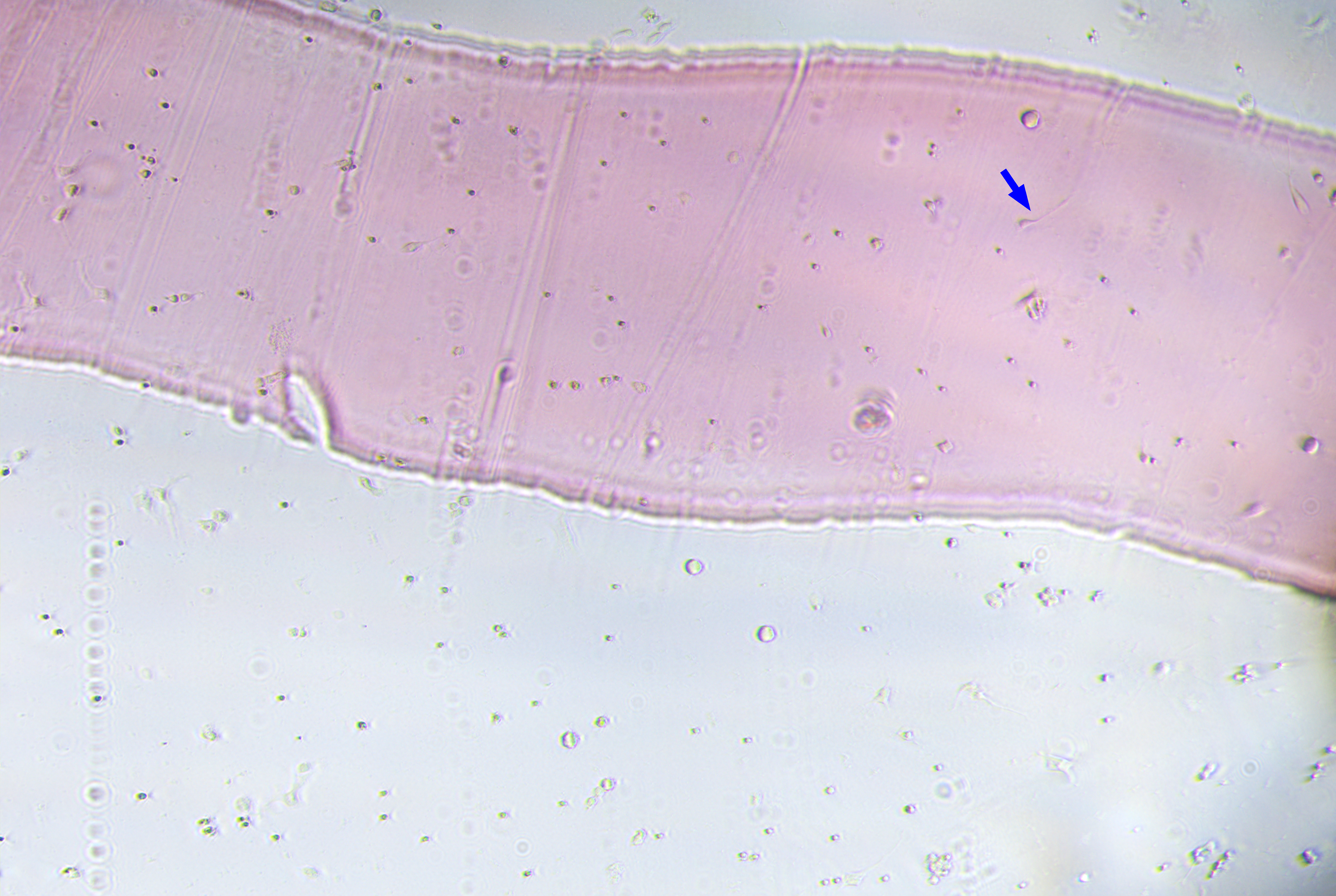

Supplement: Supplementary file 7 — Source data Fig. 1 [file 44318_2024_326_MOESM7_ESM.zip › Fig.1/Fig. 1E/Upper_0 h.tif]

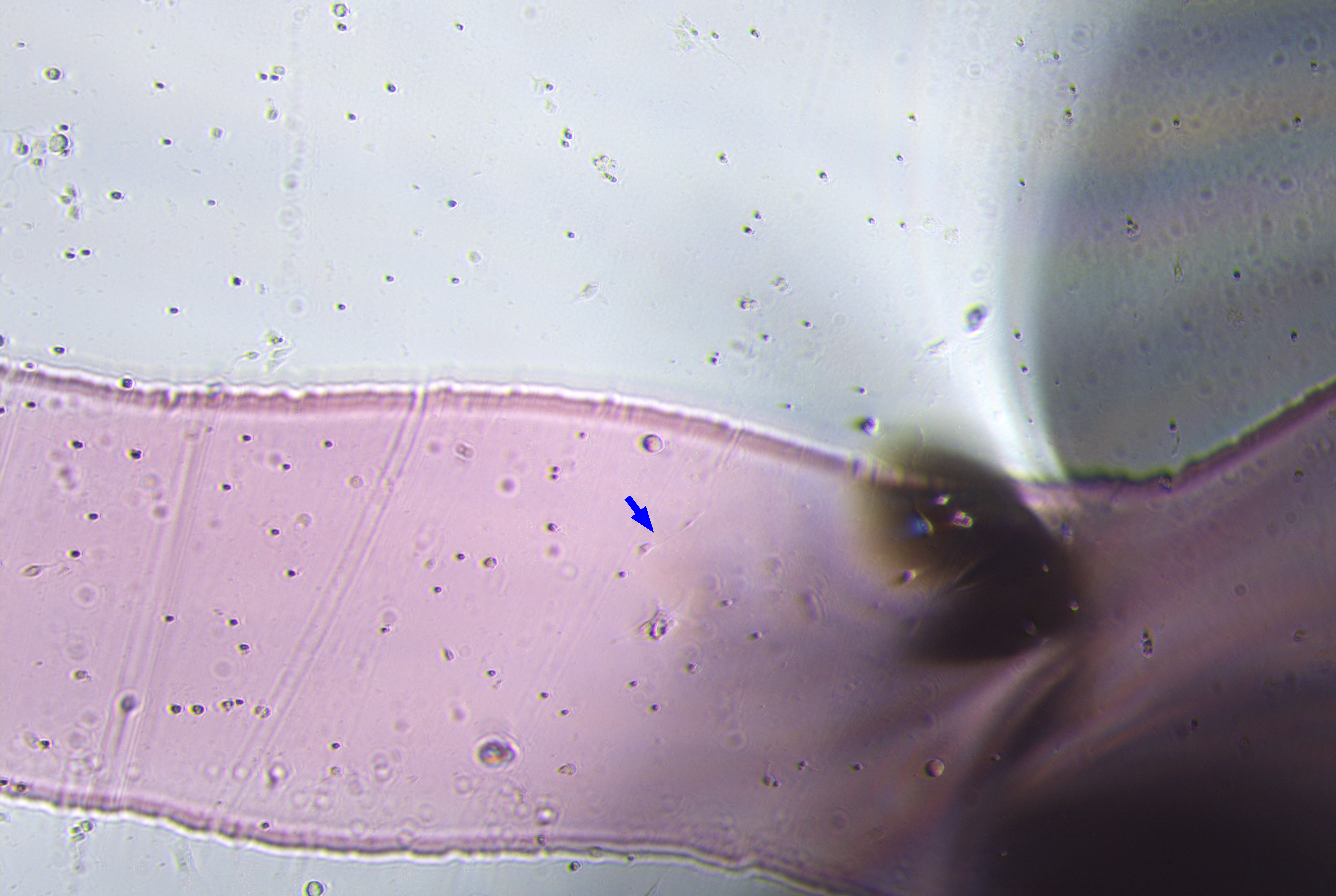

Supplement: Supplementary file 7 — Source data Fig. 1 [file 44318_2024_326_MOESM7_ESM.zip › Fig.1/Fig. 1E/Upper_1 h.tif]

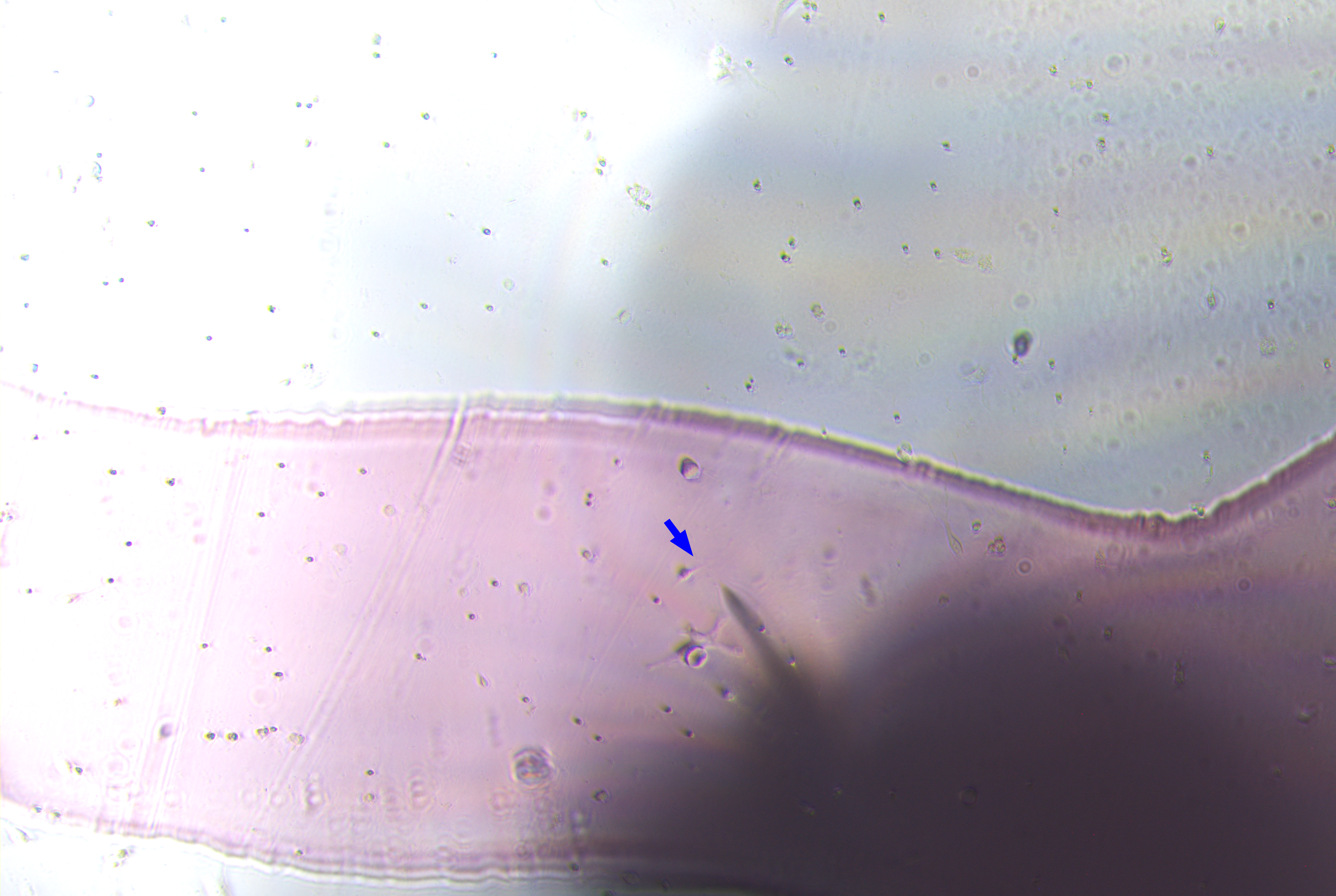

Supplement: Supplementary file 7 — Source data Fig. 1 [file 44318_2024_326_MOESM7_ESM.zip › Fig.1/Fig. 1E/Upper_2 h.tif]

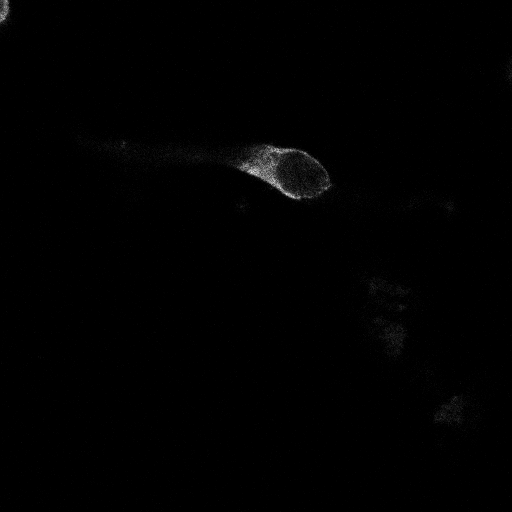

Supplement: Supplementary file 9 — Source data Fig. 3 [file 44318_2024_326_MOESM9_ESM.zip › Fig.3/Fig. 3D/Doublecortin.tif]

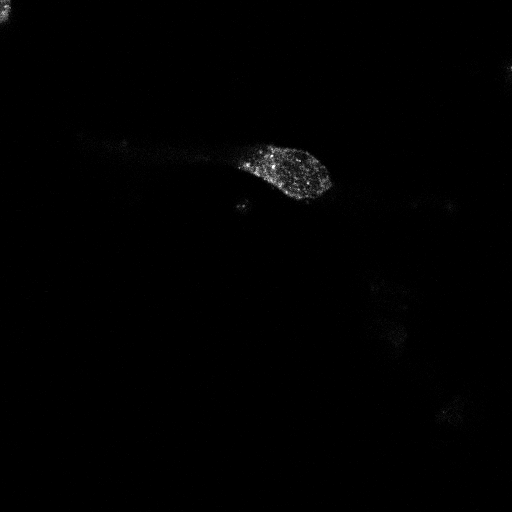

Supplement: Supplementary file 9 — Source data Fig. 3 [file 44318_2024_326_MOESM9_ESM.zip › Fig.3/Fig. 3D/Tmem63b.tif]

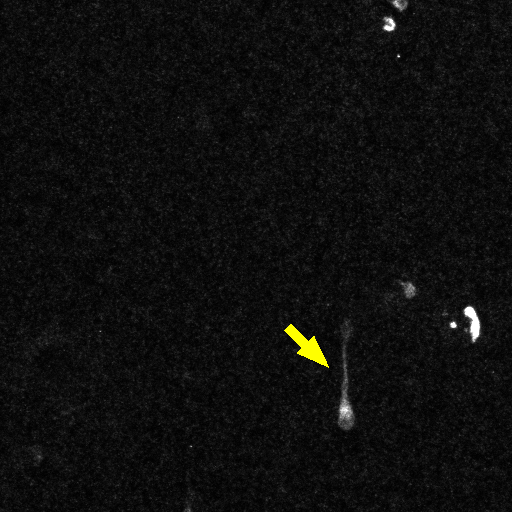

Supplement: Supplementary file 10 — Source data Fig. 4 [file 44318_2024_326_MOESM10_ESM.zip › Fig.4/Fig. 4A/Control RNAi_MLC.tif]

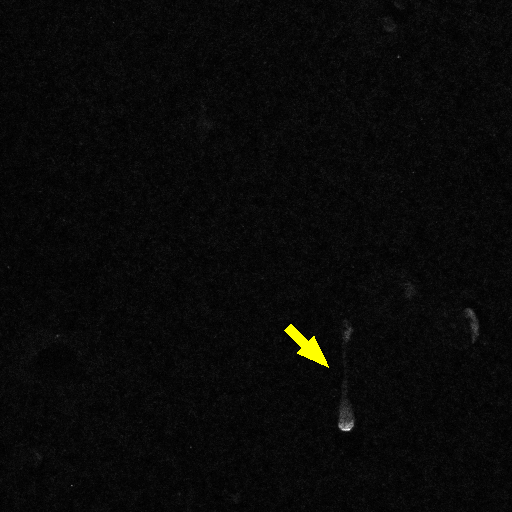

Supplement: Supplementary file 10 — Source data Fig. 4 [file 44318_2024_326_MOESM10_ESM.zip › Fig.4/Fig. 4A/Control RNAi_pMLC.tif]

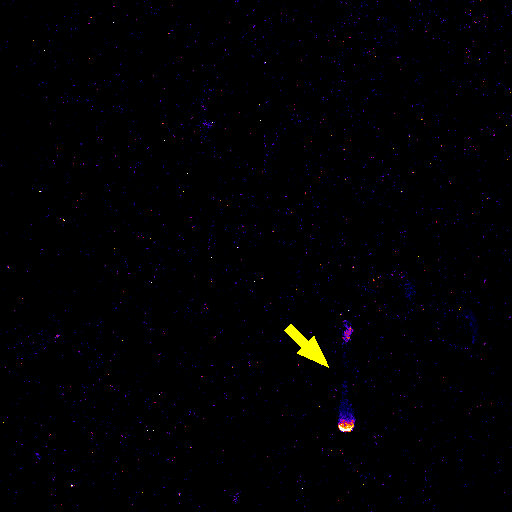

Supplement: Supplementary file 10 — Source data Fig. 4 [file 44318_2024_326_MOESM10_ESM.zip › Fig.4/Fig. 4A/Control RNAi_Ratio of pMLC and MLC.tif]

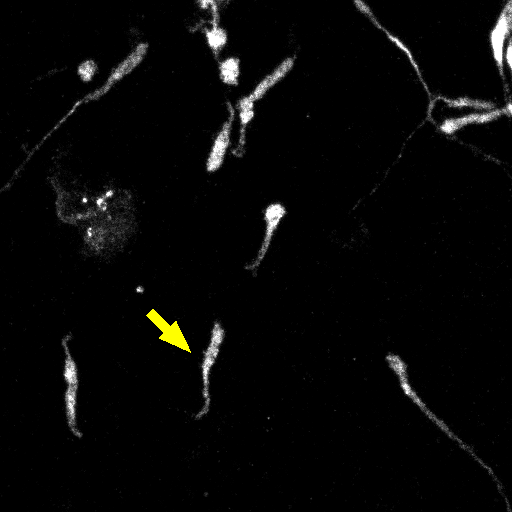

Supplement: Supplementary file 10 — Source data Fig. 4 [file 44318_2024_326_MOESM10_ESM.zip › Fig.4/Fig. 4A/Tmem63b RNAi + Tmem63br_MLC.tif]

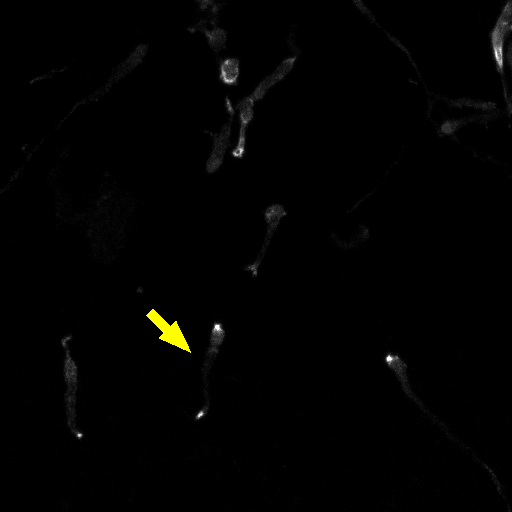

Supplement: Supplementary file 10 — Source data Fig. 4 [file 44318_2024_326_MOESM10_ESM.zip › Fig.4/Fig. 4A/Tmem63b RNAi + Tmem63br_pMLC.tif]

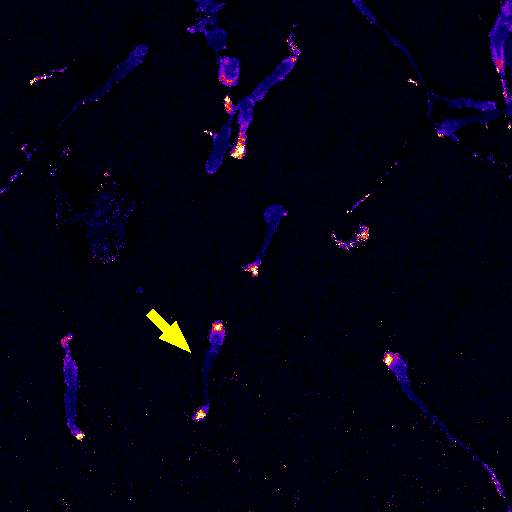

Supplement: Supplementary file 10 — Source data Fig. 4 [file 44318_2024_326_MOESM10_ESM.zip › Fig.4/Fig. 4A/Tmem63b RNAi + Tmem63br_Ratio of pMLC and MLC.tif]

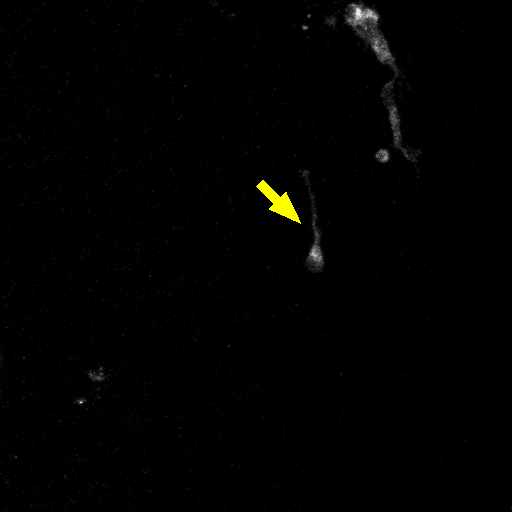

Supplement: Supplementary file 10 — Source data Fig. 4 [file 44318_2024_326_MOESM10_ESM.zip › Fig.4/Fig. 4A/Tmem63b RNAi_MLC.tif.tif]

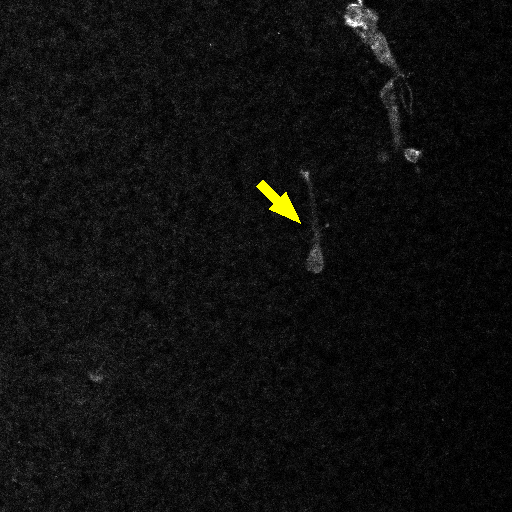

Supplement: Supplementary file 10 — Source data Fig. 4 [file 44318_2024_326_MOESM10_ESM.zip › Fig.4/Fig. 4A/Tmem63b RNAi_pMLC.tif]

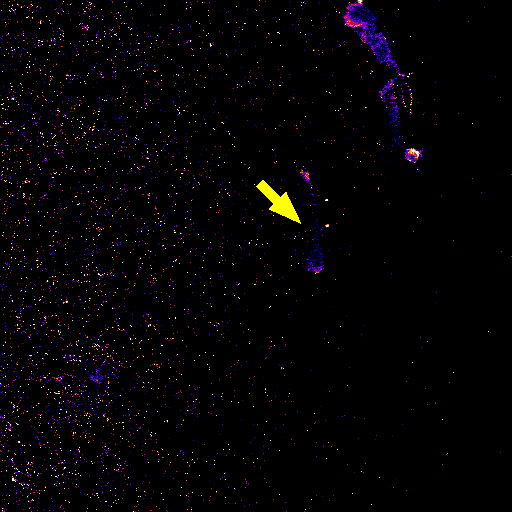

Supplement: Supplementary file 10 — Source data Fig. 4 [file 44318_2024_326_MOESM10_ESM.zip › Fig.4/Fig. 4A/Tmem63b RNAi_Ratio of pMLC and MLC.tif]

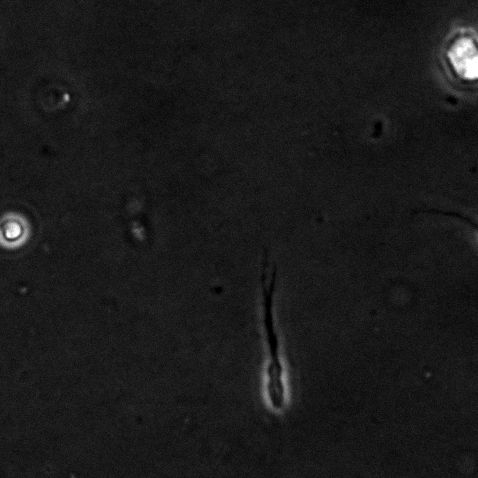

Supplement: Supplementary file 11 — Source data Fig. 5 [file 44318_2024_326_MOESM11_ESM.zip › Fig.5/Fig. 5A/Time-lapse phase-contrast images_Control microRNA.tif]

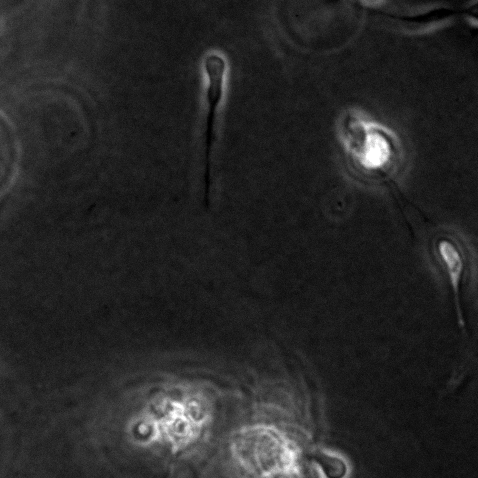

Supplement: Supplementary file 11 — Source data Fig. 5 [file 44318_2024_326_MOESM11_ESM.zip › Fig.5/Fig. 5A/Time-lapse phase-contrast images_Tmem63b microRNA + Tmem63br.tif]

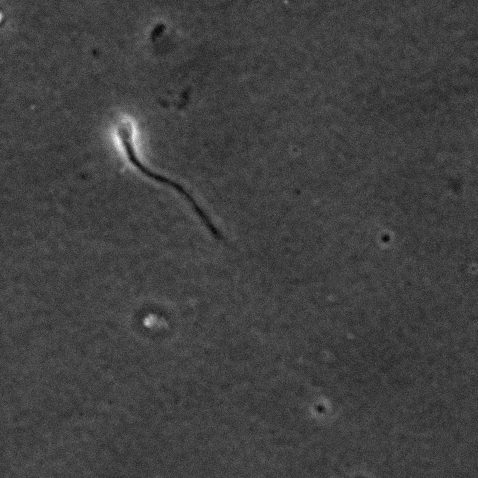

Supplement: Supplementary file 11 — Source data Fig. 5 [file 44318_2024_326_MOESM11_ESM.zip › Fig.5/Fig. 5A/Time-lapse phase-contrast images_Tmem63b microRNA.tif]
